# Frontal lead in preterm functional brain maturation

**DOI:** 10.1016/j.dcn.2026.101830

**Published:** 2026-10-02

**Authors:** Johannes Mader, Manfred Hartmann, Katrin Klebermass-Schrehof, Tobias Werther, Anastasia Dressler, Johannes Koren, Gregor Kasprian, Victor Schmidbauer, Renate Fuiko, Lisa Oberdorfer, Nadine Pointner, Angelika Berger, Tilmann Kluge, Vito Giordano

**Affiliations:** a Division of Neonatology, Pediatric Intensive Care and Neuropediatrics, Department of Pediatrics and Adolescent Medicine, Comprehensive Center for Pediatrics (CCP), Medical University of Vienna, Vienna, Austria; b Center for Health & Bioresources, AIT (Austrian Institute of Technology), Vienna, Austria; c Member of the ERN EpiCare, Lyon, France; d Karl Landsteiner Institute for Clinical Epilepsy Research and Cognitive Neurology, Vienna, Austria; e Department of Neurology, Clinic Hietzing, Vienna, Austria; f Department of Radiology, Medical University of Vienna, Austria

**Keywords:** Deep learning, EEG, Functional brain maturation, Spatial EEG development, Preterm infants

## Abstract

Understanding how brain maturation unfolds in preterm neonates is critical for both neuroscience and clinical care. Structural MRI suggests that the frontal cortex develop late, yet EEG offers a direct window into functional activity that follows a different trajectory. We analysed 211 EEG recordings from 62 preterm neonates (25–40 weeks postmenstrual age, PMA) using convolutional neural networks trained on single channels to estimate functional brain age (FBA). We introduced the spatial difference in FBA (sd_FBA) metric, which quantifies the relative maturity of each channel compared with all others within a recording. sd_FBA revealed a consistent maturation gradient, frontal regions exhibited the highest sd_FBA, followed by central regions, whereas occipital and temporal regions showed lower values. With increasing PMA, central maturation converged with frontal values, while occipital regions remained significantly lower. Functional maturation in preterm infants follows a clear frontal-occipital gradient, in contrast to structural MRI findings. The sd_FBA metric provides a novel, normalized index for tracking regional maturation, offering a new tool to understand functional brain development and potentially improve neurodevelopmental risk stratification.

## 1 Introduction

Preterm birth occurs during a period of exceptionally rapid brain development, when cortical circuits, early connectivity patterns, and spontaneous electrical activity change from week to week (Doria et al., 2010, Kostović and Jovanov-Milosević, 2006). During this developmental window, maturation of the human fetal brain follows a hierarchical and regionally specific sequence, encompassing neurogenesis, synaptogenesis, dendritic growth, and the emergence of spontaneous activity transients that precede the formation of stable functional networks (Kostović and Jovanov-Milosević, 2006, Vanhatalo and Kaila, 2006). Serial EEG recordings provide a direct view into functional maturation showing progressive reductions in discontinuity and increases in rhythmic activity across preterm development(Britton et al., 2016). Quantitative spectral analysis reveals a developmental decrease in delta and theta power alongside increases in alpha and beta bands (Niemarkt et al., 2011, Pavlidis et al., 2017, Stevenson, 2016). Notably, frontal and central channels had lower delta powers, especially for lower post menstrual ages (PMAs). Aperiodic spectral analysis demonstrates a developmental decrease in the aperiodic exponent, alongside regionally heterogeneous patterns during the neonatal period, with relatively lower frontal values(Schaworonkow and Voytek, 2021; Stanyard et al., 2024). Similar results have been observed in a similar studies in preterm infants from 27 to 37 weeks PMA on EEG connectivity (Meijer et al., 2016, Meijer et al., 2014). These findings align with results from a recent studies by Varley et al. (2025) on synergy in the preterm EEG and Stevenson et al. (2025) on differences in functional brain age (FBA), demonstrating a frontal lead in functional synergy and FBA (Stevenson et al., 2025, Varley et al., 2025).

In recent years studies developed algorithms to estimate the FBA of the preterm EEG. They used machine-learning algorithms trained to predict the PMA of preterm infants without major neurological conditions based on EEG. Such models can predict an individual infant’s PMA with mean absolute errors of less than one week(Ansari et al., 2024; Li et al., 2025; Mader et al., 2024; Pillay et al., 2020; Stevenson, 2023). An estimate of PMA can be taken as a measure for FBA, also called EEG maturational age (EMA) in other publications. Li et al. (2025) recently demonstrated that frontal and frontal-temporal channel combinations yield the most accurate FBA estimates, although spatial differences in predicted FBA values were not explored (Li et al., 2025). While this study highlighted differences in estimation performance across channels, it did not analyse the spatial differences in estimated FBA values.

Such spatial differences between the FBA and the emergence of synergy in different channels were analysed in a recent high density EEG study (Stevenson et al., 2025, Varley et al., 2025). Their findings provide further evidence for gradients in spatial FBA in the developing brain, showing that frontal regions appear functionally older than posterior areas in infants aged 36–42 weeks PMA.

Building on these insights, our study investigated spatial variations in FBA across a wide range of extremely preterm development with a focus on the temporal evolution. We compared FBA estimates across individual EEG channels using end-to-end CNN models trained directly on single-channel EEG to generate channel-specific FBA estimations. Based on these models we introduced the spatial differences in FBA (sd_FBA) metric. By analysing how sd_FBA trajectories evolve from extremely preterm to term-equivalent age, we aim to identify a spatial functional maturational gradient and its transformation over time.

## 2 Methods

In our study we utilized data from a prospectively collected preterm EEG dataset, which consists of EEG recorded at the Comprehensive Center of Pediatrics, Medical University of Vienna, between 2011 and 2017, with approval from the local ethics committee (EK Nr: 67/2008).

For this study, we trained for each single EEG-channel an individual machine-learning model to predict the PMA of preterm neonates to estimate the EEG maturational age. After evaluating all trained models on all other single EEG-channels we investigated how the FBA changes on a spatial level with maturation.

### 2.1 Data acquisition

The recordings were obtained using nine scalp electrodes at a sampling rate of 256 Hz with the Brain Quick / ICU EEG system (MicroMed, Treviso, Italy).

We excluded infants who died before the one-year follow-up examination or who had severe neurological disorders (status epilepticus, periventricular leukomalacia (PVL), intraventricular haemorrhage (IVH) grade three or higher(Vohr and Ment, 1996)). Since sedation influences the EEG, we further excluded episodes which started within 6 h after administration of sedative medication (Fentanyl, Midazolam, Morphine HCl)(Bernet et al., 2010; Obeid and Tsuchida, 2016).

#### 2.1.1 EEG preprocessing

Preprocessing was performed similarly to previously published literature(Stevenson et al., 2017; Stevenson, 2016). EEG was recorded on the electrodes Fp1, Fp2, C3, C4, T3, T4, O1, and O2, with a common reference electrode at Fpz. Bipolar channels were computed for all adjacent electrodes resulting in the 13 bipolar channels Fp1-Fp2, Fp1-T3, Fp1-C3, Fp2-C4, Fp2-T4, C3-C4, T4-C4, T3-C3, C3-O1, C4-O2, O1-O2, T3-O1, and T4-O2.

The signal was filtered using a 0.5–32 Hz bandpass filter (Butterworth, 5th order) and then down sampled from 256 Hz to 64 Hz for data compression. Periods of 1 s of abnormally high absolute amplitude (> 300 μV) or periods of 60 s with more than 90% of the absolute amplitude below 1 μV, were classified as artifact and thus excluded from training and evaluation.

The bipolar montage was selected for the analysis because it provides estimates of the tangential electric field halfway between the electrodes, and reduces contributions from activity shared across electrodes, including common-reference effects and spatially widespread signals (Yao et al., 2019). We repeated the complete analysis using monopolar channels, including independent training of all single-channel CNN models, see Supplementary Material A.7.

### 2.2 FBA estimation

FBA is a non-observable, latent construct that reflects the degree of EEG maturation of the preterm brain. We estimated FBA using an end-to-end CNN based on our previously published medium-sized architecture (Mader et al., 2026). Each model used a single-channel EEG 144 s segments at 64 Hz and consisted of four sequential convolution-pooling blocks followed by a regression output predicting PMA. The CNN generated age estimates with 1 Hz, which were aggregated using their median to obtain one FBA estimate per recording. The network contained 53 793 trainable parameters. Model training and evaluation were performed using eight-fold cross-validation at the patient level, ensuring that recordings from the same infant were never included in both training and validation sets. Further details of training, validation splits, and parameter settings provided in Supplementary Material A2. In total 351.54 h of EEG were used for training and validation.

### 2.3 Computing spatial differences in FBA (sd_FBA)

Direct comparison of channel-wise FBA estimates is complicated by two factors. First, the absolute FBA of all channels is strongly determined by the PMA of the infant, but our primary interest is the relative maturity of one EEG channel compared to the remaining channels within the same recording. Second, predictions from models trained on anatomically or functionally similar channels are highly correlated and should therefore not receive disproportionate weight. We introduced sd_FBA to address both issues. In simple terms, sd_FBA describes how much older or younger the EEG of one channel appears relative to the weighted average of the other channels in the same recording. A positive sd_FBA therefore indicates relatively advanced EEG maturation, whereas a negative value indicates relatively delayed maturation.

To investigate spatial differences in FBA, we trained separate CNN models for each EEG channel, enabling direct comparison of FBA estimates across scalp locations. We define FBA_chE,chT_ as the FBA of channel chE estimated by the model trained on the channel chT, see Fig. 1. This value is an estimate of how developed the EEG of channel chE is based on the data from channel chT. Based on these FBA estimations we define the spatial FBA (sFBA) as

(2) $${\mathrm{sFBA}}_{ch_{E}}≔\sum_{\mathrm{chT}}w_{j}{\mathrm{FBA}}_{ch_{E},ch_{T}},$$

with weights *w*_*T*,_ see Fig. 1d. This is a measure for how developed channel ch_E_ is on average based on the estimations from all other channel-models.

**Fig. 1:**
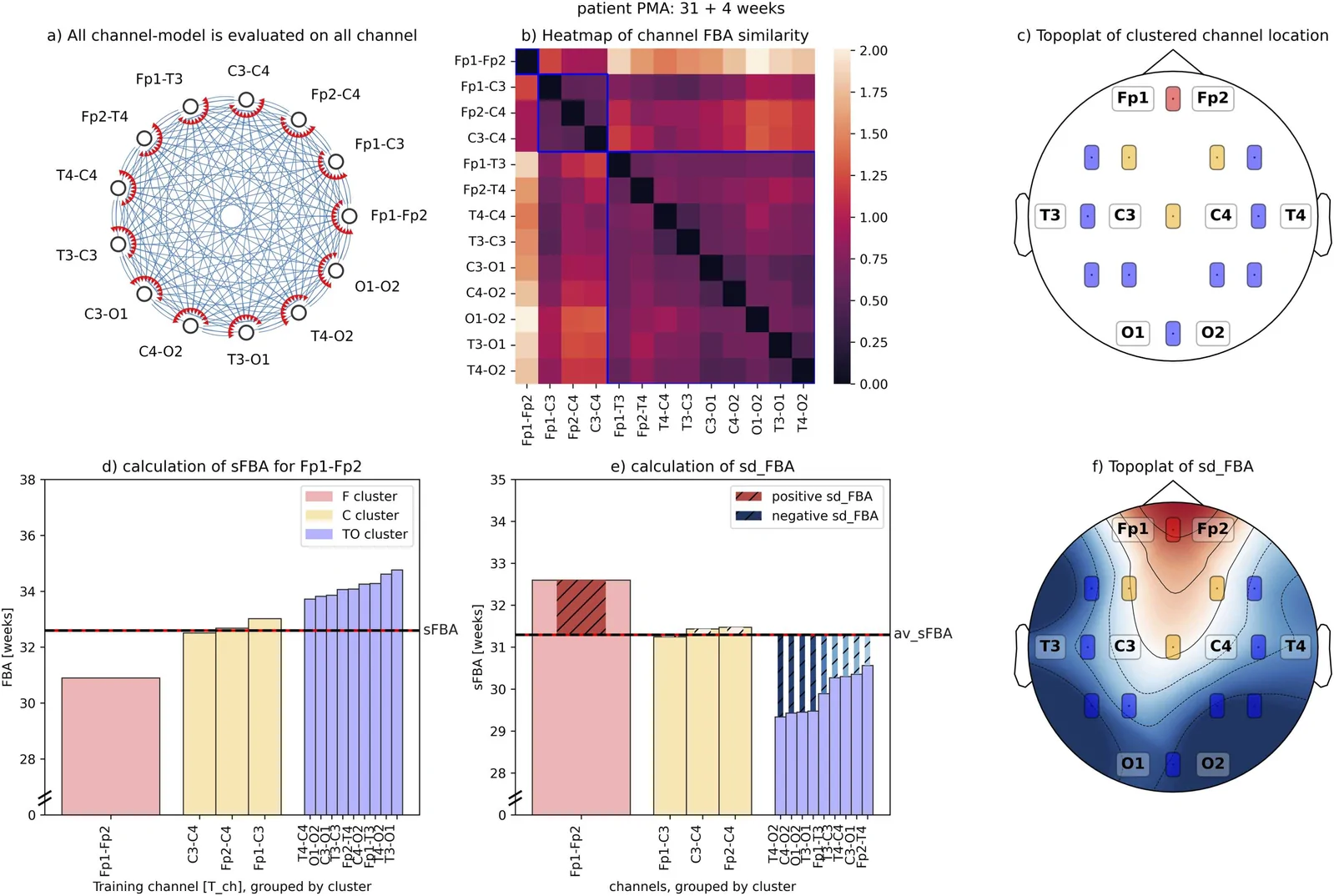
The process of calculating the sd_FBA value. *a) All single-channel models are evaluated on all channels. b) Heatmap visualizing channel-similarity in FBA estimation and clustering. c) Spatial location of the three clusters. d) An example of FBA for the channel Fp1-Fp2 from all single-channel models and the sFBA values as the weighted average. PMA at time of recording was 31 + 4 weeks. e) sFBA values for all channels and the sd_FBA values as the differences between the sFBA and the weighted average for the same recording as d). f) Topoplot visualizing the sd_FBA values from e) spatially.*

For the averaging we did not use the arithmetic mean ($w_{T}=\frac{1}{N}\;)$ as this may disproportionately represent similar values, leading to biased results (Rousseeuw and Leroy, 1987). To address this, we cluster channels with similar FBA estimations together by calculated the mean absolute estimation difference between all pairs of channels across all evaluated recordings, see Fig. 1b. A smaller value indicates greater similarity between two channels. The channels were then grouped using the K-means clustering algorithm, with the optimal number of clusters #CL determined via the standard elbow method(MacQueen, 1967). This led to the cluster weights

$$w_{T}≔\frac{1}{\#\mathrm{CL}\cdot \#Cl_{T}},$$

where #Cl_T_ is the number of channels in the cluster containing channel ch_T_.

The final sd_FBA corrects for the weighted average across all channels to ensure that the weighted average of all channels is zero,

(3) $${\mathrm{sd}\_\mathrm{FBA}}_{ch_{i}}≔{\mathrm{sFBA}}_{ch_{i}}-\sum_{j=0}^{N}w_{j}{\mathrm{sFBA}}_{ch_{j}},$$

see Fig. 1e. Hence, sd_FBA measures how many weeks on average channel ch_E_ is ahead in sFBA compared to all other channels.

#### 2.3.1 Complementary analysis of sd_FBA with different datasets

We performed two complementary analyses. First, we replicated the sd_FBA analysis on an independent multichannel EEG dataset from Helsinki (Stevenson et al., 2019). Second, we recalculated sd_FBA using single-channel FBA models independently trained on EEG recorded with aEEG monitors (see Supplementary Material A.5 and A.6).

### 2.4 Statistical evaluation of the spatial maturation

Spatial differences in functional brain maturation were assessed using the defined sd_FBA metric. For the frontal-occipital axis, bipolar channels were ordered anatomically from frontal to occipital, and for the lateral axis from temporal to central regions. Associations between sd_FBA and spatial position were quantified using Pearson correlation coefficients, computed across the full PMA range and within predefined PMA bins (25–28, 28–32, 32–36, and 36–41 weeks). To characterise individual-level spatial maturation, frontal–occipital (F–O) and temporal–central (T–C) gradients were calculated for each recording as the slope of a linear regression fitted to sd_FBA values across spatial positions. Developmental changes in these gradients were assessed by correlating individual gradient values with PMA using Pearson correlation. Statistical significance was defined as two-sided p < 0.05. All analyses were performed in Python using numpy, pandas and scipy.stats.

## 3 Results

The prospective dataset consists of in total of 70 patients with 246 conventional EEG recordings. Patients were born between 23 and 28 weeks of gestational age (GA), and their EEG recordings were collected during 24- and 42-weeks PMA. A total of 8 patients were excluded based on exclusion criteria (2 had PVL, 6 severe IVH), leaving a final dataset of 62 patients and 211 recordings taken between 25- and 40-weeks PMA. Description of the dataset and the excluded patients is shown in Table 1.

**Table 1:** Description of the dataset.

	unit	included	excluded
patients	n	62	8
files	n	211	31
EEG [h]	µ [σ]	3.0 [1.0]	1.6 [1.3]
GA	µ [σ]	25.9 [1.5]	25.6 [0.2]
PMA	µ [σ]	32.2 [3.4]	30.6 [5.2]
birth weight	µ [σ]	730.0 [171.4]	748.0 [168.0]
APGAR 5	µ [σ]	7.8 [2.2]	6.1 [4.5]
BSID 2 y.	µ [σ]	87.5 [18.7]	68.0 [13.0]
SEX [female]	%, [n]	50.0% [31]	50.0% [4]
IVH	%, [n]	17.7% [11]	87.5% [7]
NEC	%, [n]	5.1% [3]	16.7% [1]
ROP	%, [n]	56.9% [33]	66.7% [4]
CLD	%, [n]	24.6% [14]	66.7% [4]

Values are expressed as mean and standard deviation (µ [σ]), or percentage with number of cases (% [n]), or absolute counts (n). GA: gestational age at birth; PMA: postmenstrual age at EEG recording; BSID 2 y.: Bayley Scales of Infant and Toddler Development cognitive score at 2 years; IVH: intraventricular hemorrhage; NEC: necrotizing enterocolitis; ROP: retinopathy of prematurity; CLD: chronic lung disease.

### 3.1 Evaluation of the individual single-channels models

All 13 models, each trained on a specific bipolar EEG channel, showed similar performance when evaluated on the same EEG channel, with predicted age difference (PAD) (=EMA-PMA) values centred around zero, as illustrated in supplementary material in Fig. B1. A one-way ANOVA test revealed no significant differences in performance across these models. All eight models trained on monopolar EEG channels showed the same behaviour and no significant differences in performance according to a one-way ANOVA. These results support the comparability of the models.

### 3.2 Computing spatial EEG maturation on a channel pair level

We examined differences in FBA across channels by evaluating the FBA of a target channel (chE) using models trained on other channels (chT), denoted as ${\mathrm{FBA}}_{\mathrm{chE},\mathrm{chT}}$. Values were close to the PMA of the recording for identical channel pairs, see Fig. 1a-c and supplementary Fig. B1. For non-identical pairs, however, systematic deviations were observed. Specifically, ${\mathrm{FBA}}_{\mathrm{chE},\mathrm{chT}}$ of the frontal channel Fp1–Fp2 estimated by the occipital model O1–O2 was consistently higher than the reverse case, see Fig. 1e

This study focuses on how FBA differs across different channels. We compared the FBA for channel chE estimated by the model trained on channel chT (${\mathrm{FBA}}_{\mathrm{chE},\mathrm{chT}}$). As mentioned in the previous section, values are centred around zero regardless of the channel, as shown in the first row of Fig. 1. This is not the case for non-identical channel pairs. FBA values of the frontal channel Fp1-Fp2 evaluated by the model of the occipital channel O1-O2 were higher than the FBA values of O1-O2 evaluated by the Fp1-Fp2 model, see Fig. 1.

In this comparison the ${\mathrm{FBA}}_{\mathrm{Fp}1-\mathrm{Fp}2,O1-O2}$ value was larger than the ${\mathrm{FBA}}_{O1-O2,\mathrm{Fp}1-\mathrm{Fp}2}$ value in all recordings. Similarly, the frontal FBA values were higher in 87% of recordings in the frontal-central channel pair Fp1-Fp2 and C3-C4, and the central FBA values were higher in 92% of recordings in the central-occipital channel pair C3-C4 and O1-O2, see Fig. 2. If we look at the overall percentage of instances in which one channel is estimated higher within a channel-pair across all recordings, the frontal Fp1-Fp2 channel reaches 96%, followed by the central C3-C4 channel with 78%, and the other central channels Fp2-C4, Fp1-C3 with 74% and 64%, respectively. Conversely, the FBA of the occipital channel O1-O2 was higher than that of the other channel in 20% of recordings and channel-pairs, see supplementary Tab B1.

**Fig. 2:**
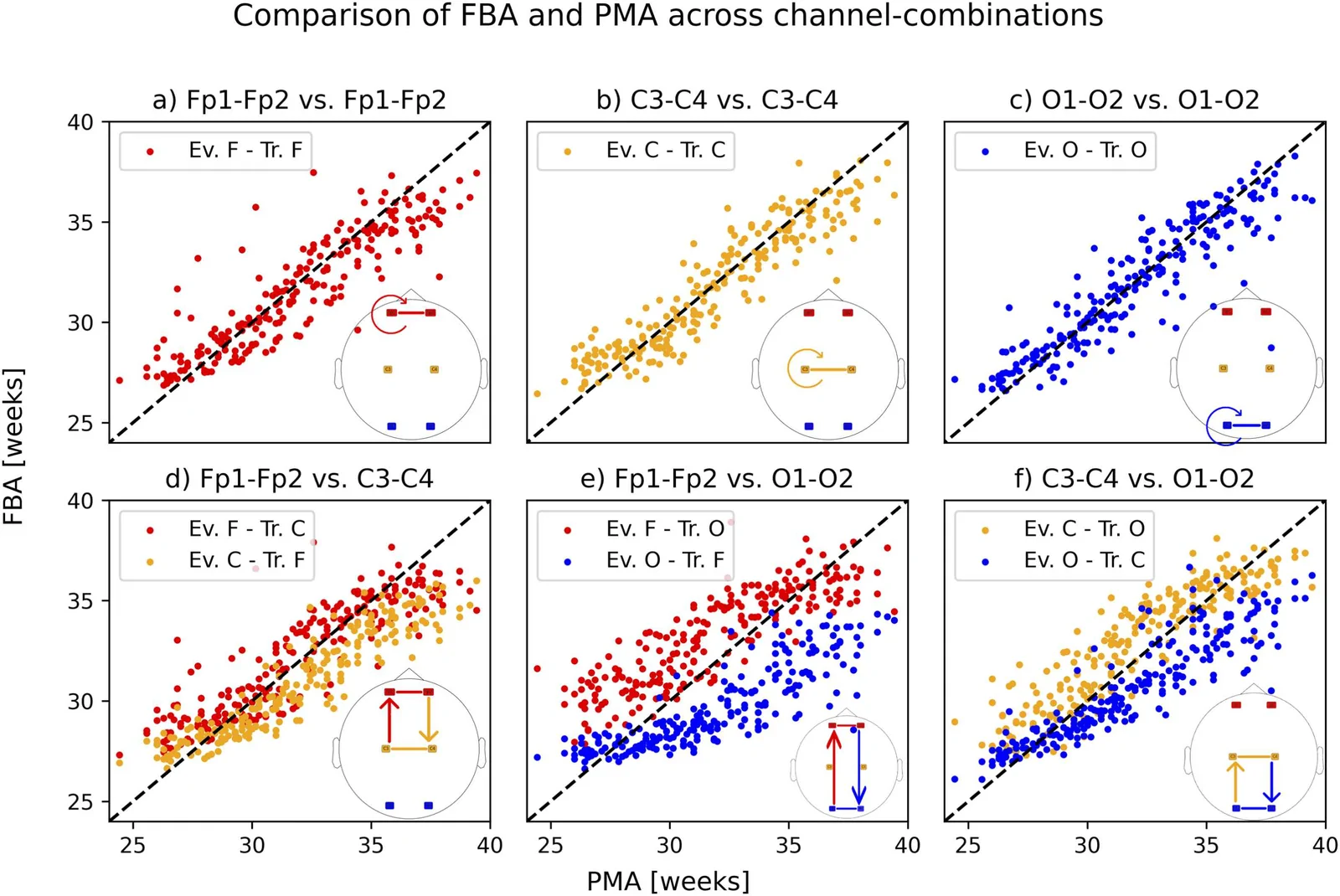
Pair wise PMA vs FBA. *Visualization of PMA values vs. FBA values for all channel pairs of the channels Fp1-Fp2, C3-C4, and O1-O2. The labelling “Ev. F, Tr. C”, for example, indicates FBA values for the frontal evaluation channel Fp1-Fp2 by the model trained on the central channel C3-C4.*

### 3.3 Clustering of similarly evolving channels

The elbow-method suggested three clusters. The first cluster consisted of only the frontal channel *Fp1-Fp2*. The second cluster included the central channels *Fp1-C3, Fp2-C4,* and *C3-C4.* The third cluster included all other channels. We therefore refer to the first cluster as the frontal cluster (F), the second cluster as the central cluster (C), and the third cluster as the temporal-occipital cluster (TO). The positions of these clusters are visualised in Fig. 3 and similarities of all channel pairs are visualized in Fig. B.2.

**Fig. 3:**
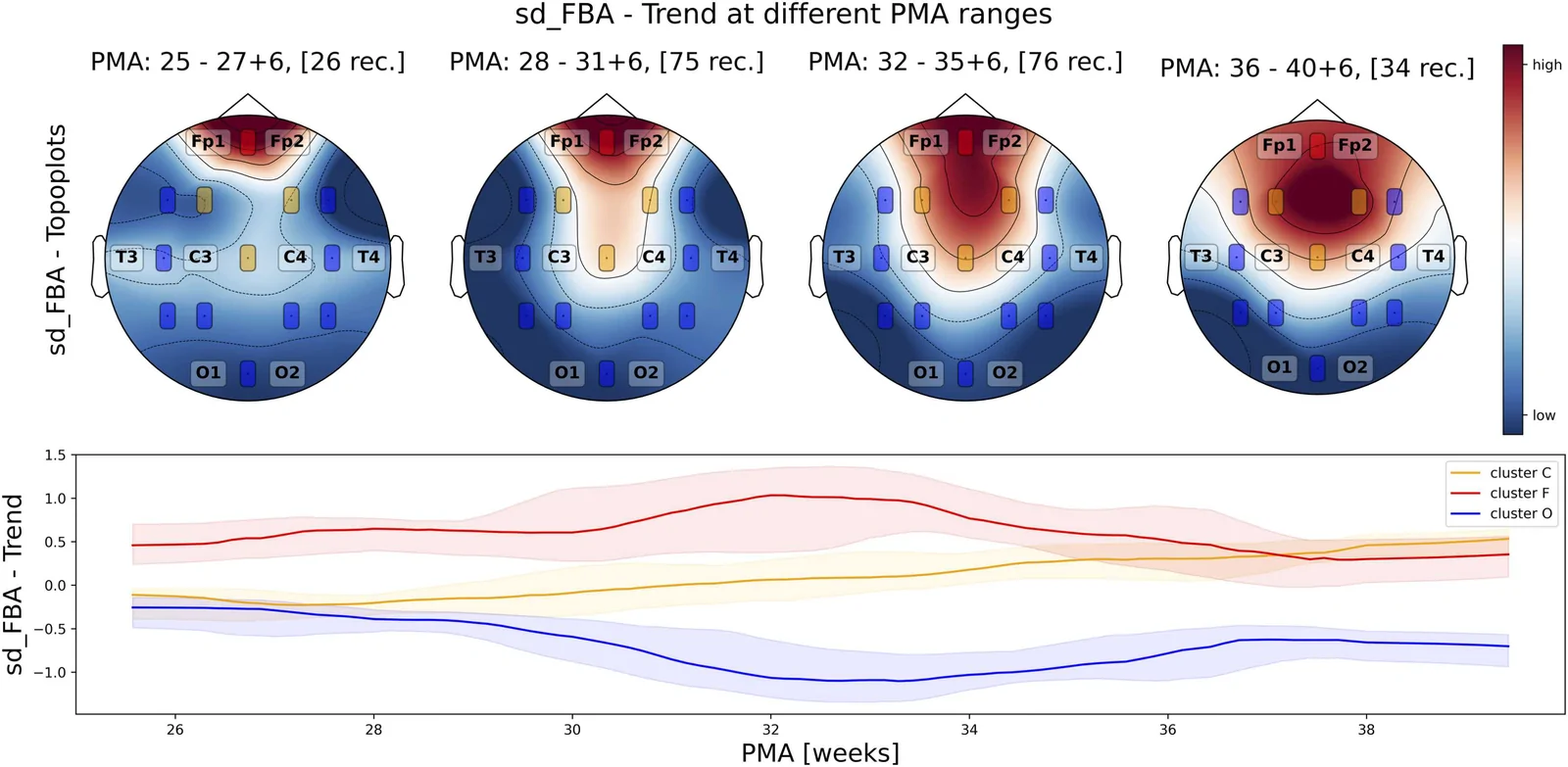
sd_FBA values spatially visualised at different PMA ranges. *Visualization of the temporal evolution of the spatial difference in FBA (sd_FBA) in four groups. The first row shows Topoplots of the spatial maturation values in four PMA groups. The second row shows the corresponding spatial maturation trend of the three clusters.*

### 3.4 Spatial differences in FBA

sd_FBA values, measuring the relative FBA of one channel compared to all others, were calculated from 93 recordings. 99% of sd_FBA values ranged between −2.95 and 1.66, with variations observed among the clusters. The frontal channel had the highest median value at 0.64 [IQR: 0.27, 1.04], the central channel C3-C4 had a median sd_FBA of 0.05 [IQR: −0.25, 0.52], the temporal channels T3-C3 and T4-C4 had a median sd_FBA of −0.49 [IQR: −0.99, −0.11], and the occipital channel O1-O1 had the lowers median sd_FBA at −1.02 [IQR: −1.45, −0.53], see supplementary Tab B2. This distribution of sd_FBA values is in accordance with the channel wise comparison in Fig. 2.

### 3.5 Evolution of spatial maturation with PMA

To evaluate how the FBA changes spatially with increasing PMA, we grouped the data into four groups (25–27 +5 weeks, 28–31 +6 weeks, 32–35 +6 weeks, and 36–40 +6 weeks). The median sd_FBA values within each PMA group were visualised in Topoplots which capture the spatial component of these values, see Fig. 3. The grouped sd_FBA values were highly symmetric from left to right across all age groups but showed a shift from frontal to occipital regions.

In the earliest age group (25–27 +6 weeks PMA), sd_FBA values were significantly higher in the frontal cluster compared to both the central and temporal-occipital clusters (p < 0.001 for both; Fig. B2). As PMA increased, sd_FBA values in the central channels rose in parallel with those in the frontal ones, resulting in a clear separation between all three clusters during 28–35 + 6 weeks PMA. By 36–40 + 6 weeks PMA, sd_FBA values in the central cluster reached levels comparable to the frontal cluster, and the difference between these two clusters was no longer significant. Nevertheless, values in both frontal and central clusters remained significantly higher than those in the occipital cluster (p < 0.001; Fig. B2).

### 3.6 Frontal-occipital gradient in sd_FBA

Across the full PMA range, sd_FBA exhibited a pronounced frontal-occipital gradient along the frontal–occipital (F–O) axis (r = −0.64, p < 0.001), with frontal channels showing the highest relative maturity and occipital channels the lowest, see Fig. 4 and B3 (first row). When stratified by PMA, this gradient was low at early ages (25–28 weeks PMA; r = −0.50, p < 0.001) and progressively strengthened with increasing maturation (28–32 weeks: r = −0.57; 32–36 weeks: r = −0.71; 36–41 weeks: r = −0.72, all p < 0.001).

**Fig. 4:**
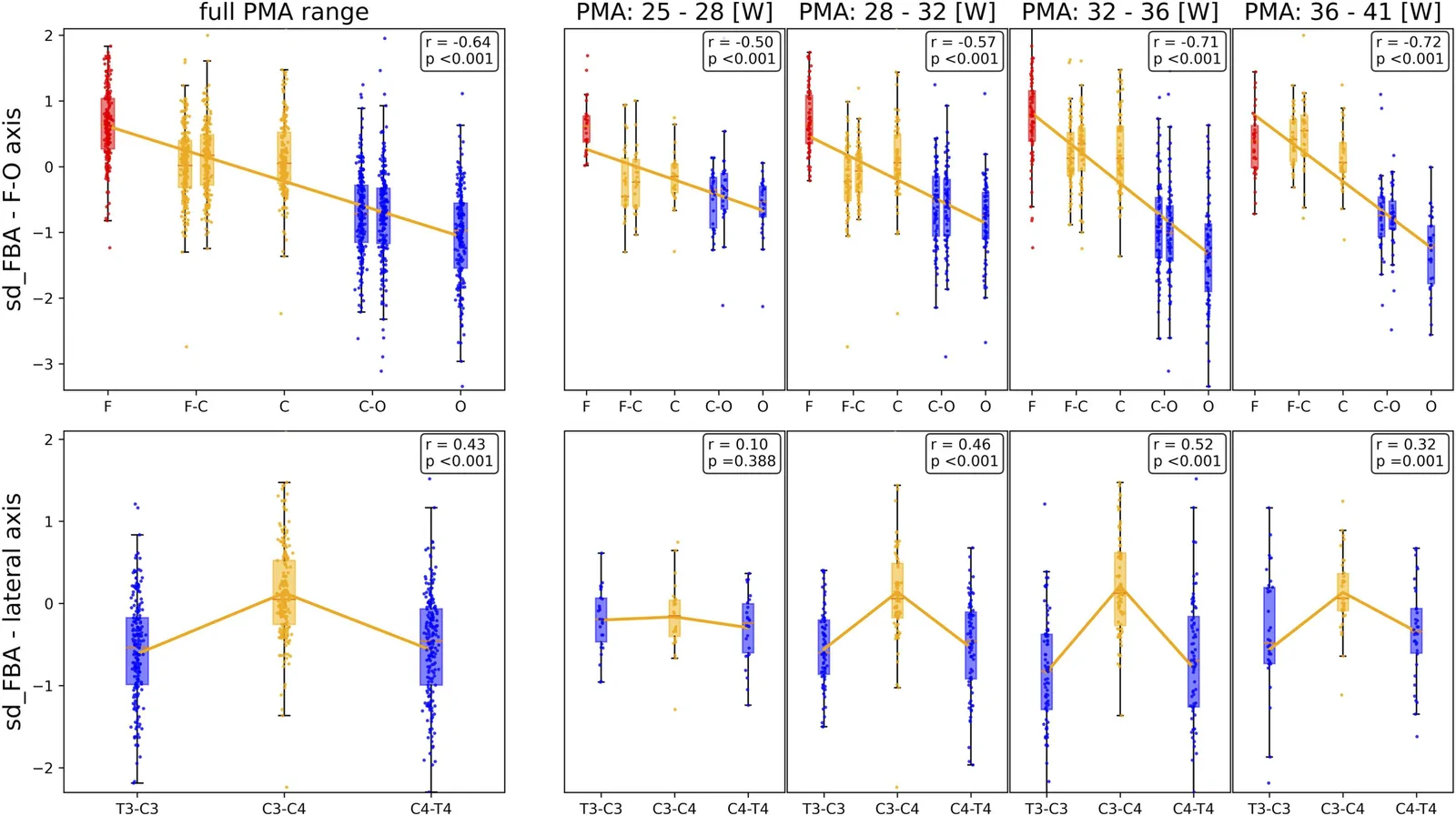
Temporal evolution of the frontal-occipital and temporal-central gradient in sd_FBA for four different PMA groups. *First row: Fronto–occipital gradient of spatial difference in functional brain age (sd_FBA) across postmenstrual age (PMA) groups. Boxplots show sd_FBA distributions for frontal (F), frontal-central (F–C), central (C), central-occipital (C–O), and occipital (O) channel within four PMA ranges. Second row: Temporal-central gradient of sd_FBA in the four age groups. Boxplots show sd_FBA distributions for the T3-C3, C3-C4, and C4-T4 channel. Orange lines indicate linear trends across the frontal-occipital axis or the lateral. Pearson correlation coefficients (r) and corresponding p-values are reported in each panel*.

96.2% of all individual F-O-gradients are negative with a median gradient of −0.38 (IQR [-0.59, −0.21]), see Supplementary Fig. B3. These individual F-O-gradients are negatively correlated with PMA (r = -0.4, p < 0.001), see Supplementary Fig. B3. The consistent increase in correlation indicates an increasingly well-defined spatial ordering of FBA along the frontal-occipital axis with advancing PMA.

Visual inspection of group-wise distributions further revealed that central regions gradually converged towards frontal sd_FBA values with increasing PMA, whereas occipital regions remained markedly delayed throughout the studied period.

### 3.7 Temporal-central gradient in sd_FBA

Along the lateral axis, sd_FBA showed a weaker and less consistent spatial organization. Across the full PMA range, a modest positive correlation was observed (r = 0.43, p < 0.001), reflecting higher sd_FBA in central (C3–C4) compared with lateral (T3–C3 and C4–T4) channels, see Fig. 4 (second row). Age-stratified analyses revealed no significant lateral gradient at the youngest ages (25–28 weeks PMA; r = −0.02, p = 0.915), whereas significant correlations emerged at intermediate PMA ranges (28–32 weeks: r = 0.53, p < 0.001; 32–36 weeks: r = 0.52, p < 0.001). At later ages (36–41 weeks PMA), the lateral gradient was attenuated and no longer statistically significant (r = 0.42, p < 0.001).

Individual T-C-gradients are mostly positive (87%) with a median gradient of 0.55 (IQR [0.20, 1.11]), see Supplementary Fig. B3. These individual T-C-gradients are only slightly positively correlated with PMA (r = -0.24, p < 0.001), see Supplementary Fig. B3.

### 3.8 Reproducing sd_FBA results on different data

Similar results were obtained using two independent EEG datasets. First, we analysed a multichannel EEG dataset from 22 preterm and term infants (36–44 weeks PMA) recorded in Helsinki (Stevenson et al., 2019) without seizure annotations. The clustering also yielded a frontal, central, and temporal-occipital cluster and sd_FBA values in the frontal and central cluster were significantly larger, similarly to our results. Since the Helsinki cohort comprised older infants within a substantially narrower PMA range, it does not allow replication of the age-dependent evolution observed during earlier preterm development (see Supplementary Material A.5). Second, we trained models on a large, independent EEG dataset collected from aEEG monitors including 180 recordings from 69 infants (see Supplementary Material A.6)(Mader et al., 2026). Third, we also replicated the entire analysis with a monopolar montage (see Supplementary Material A.7).

All three supplementary analyses yielded similar clustering of spatial EEG maturation values and comparable temporal sd_FBA trends across the three clusters, further supporting the robustness and generalizability of our findings.

## 4 Discussion

In this study, we investigated the spatial organization of FBA in preterm infants using a machine-learning–based EEG FBA framework. Our spatial FBA analysis reveals a highly ordered pattern of regional FBA, characterized by earlier maturation in frontal EEG regions and robust spatial gradients along both the frontal–occipital and temporal–central axes. Across the entire cohort, frontal channels consistently exhibited higher predicted FBA than more posterior regions at equivalent PMA, indicating a pronounced frontal lead in early functional development. Notably, these gradients were already present at low PMA and became more pronounced with advancing maturation, suggesting that spatial differentiation of functional maturation is an intrinsic feature of early cortical development rather than a late-emerging phenomenon.

The sd_FBA metric, which quantifies the relative functional maturity of each EEG channel with respect to all others within the same recording, further clarified these spatial patterns. Frontal regions showed the highest relative maturity at early PMA, while central regions progressively converged toward frontal values around term-equivalent age. In contrast, occipital regions remained consistently delayed throughout the studied developmental window. These findings provide a quantitative and spatially resolved description of FBA and demonstrate that maturation proceeds in a structured and hierarchical manner. Importantly, the observed gradients were reproducible across independent neonatal datasets and align with recent publications in this area (Stevenson et al., 2025, Varley et al., 2025). Furthermore, these findings align with previous research demonstrating progressive spectral power changes with PMA, where frontal channels exhibit the most pronounced early maturation(Andriessen, 2011; Meijer et al., 2016, Meijer et al., 2014).

Our results are in close agreement with recent work by Varley et al. (2025) and Stevenson et al. (2025), who reported advanced functional maturation of frontal regions in preterm infants. Varley et al. demonstrated that frontal regions lead the early development of large-scale functional brain networks between 33- and 45-weeks PMA, forming a synergistic scaffold that supports integrative, higher-order interactions prior to the maturation of more posterior and sensory regions. In this framework, frontal cortices become functionally connected earlier, while the integration of occipital and other sensory areas emerges more gradually. Stevenson et al. (2025) used the same large dataset of 267 high density 128-electrode EEG recordings between 33- and 44-weeks PMA. They used a similar approach to our sd_FBA, training FBA-model for each of the 44 parcels of the cortical surface and evaluating them on other parcels. Thereby calculating the ΔFBA value which is similar but not identical to our sd_FBA, see supplementary material A.4 for more information and a clear breakdown of the differences.

While the overall spatial patterns are highly consistent, we additionally identified a significant relationship between the strength of these gradients and PMA, which was not reported in the work by Stevenson et al. This difference is likely explained by methodological and cohort-related factors. First, sd_FBA and ΔFBA, while conceptually related, differ in normalization and sensitivity to relative channel-wise differences, which may influence the detection of age-dependent effects. Second, our dataset spans a broader PMA range, extending into extremely preterm ages, where spatial differentiation of functional maturation appears to change most rapidly. By capturing this early developmental window, our analysis may be particularly sensitive to dynamic maturation processes that are less apparent in narrower and higher PMA ranges.

### 4.1 Divergence between functional and structural development

Our findings indicate a dissociation between functional and structural maturation during the late preterm to term-equivalent period. Although structural neurodevelopmental models consistently describe delayed maturation of frontal regions, which is characterized by later myelination and slower development of intra-frontal and frontal–subcortical pathways, we observe comparatively advanced functional organization in frontal cortical areas between 24 and 40 weeks PMA (Anjari et al., 2007, Batalle et al., 2017, Buyanova and Arsalidou, 2021, Wang et al., 2019).

This apparent discrepancy reflects the hierarchical and regionally heterogeneous nature of fetal brain development, in which functional maturation does not simply mirror anatomical growth(Kostović and Jovanov-Milosević, 2006; Kostović and Judaš, 2010). While anatomical factors related to skull and fontanelle development may modestly influence regional EEG amplitudes in early life (Flemming et al., 2005, Gargiulo et al., 2015), the consistency of the observed spatial patterns across datasets and analyses argues against a predominantly biophysical explanation.

A more plausible interpretation is that functional maturation is strongly activity-dependent and accelerates during the third trimester. Large-scale functional networks, including frontoparietal and default-mode networks, are already detectable by approximately 32–36 weeks PMA and show rapidly increasing synchronization toward term (Doria et al., 2010, He and Parikh, 2016, Hu et al., 2021). Thus, frontal circuits may achieve early functional integration despite ongoing structural immaturity. From a systems perspective, this aligns with evidence that the late-gestational brain transitions toward more flexible and metastable network dynamics, with frontal associative regions acting as early hubs of integrative processing (Champaud et al., 2025).

### 4.2 Complementary analyses of sd_FBA

The spatial patterns identified in this study should be interpreted as topographic differences in EEG-derived FBA rather than as precise localization of maturation to underlying cortical regions. Scalp EEG reflects activity generated by distributed neural sources. To assess the robustness of the observed spatial organization, we performed several complementary analyses. First, we repeated the complete analysis using monopolar EEG. This analysis reproduced the principal spatial patterns obtained with the bipolar montage (Supplementary Section A.7), indicating that the findings are not dependent on the selected EEG derivation. Second, comparable spatial patterns were obtained in an independent multichannel EEG dataset from Helsinki, although its narrower and older PMA range did not allow replication of the developmental trajectory observed in the primary cohort (Supplementary Section A.5). Finally, recalculating sd_FBA using FBA models independently trained on EEG from a separate aEEG dataset yielded comparable spatial organization (Supplementary Section A.6). Together, these complementary analyses demonstrate that the principal topographic organization of EEG-derived FBA is robust across EEG montages, datasets, and independently trained FBA models.

### 4.3 Validity of channel comparison

Since EEG characteristics vary across scalp locations, maturational trajectories may differ between channels, raising concerns about the direct comparability of FBA estimates derived from heterogeneous electrodes (Andriessen, 2011, Li et al., 2025). However, channel-wise FBA comparisons are supported by two observations. First, all single-channel CNN models showed comparable performance on their respective channels, with prediction errors centred around zero and no significant differences across bipolar channels (one-way ANOVA; p > 0.05). Second, we observed strong pairwise consistency which was most pronounced between the frontal (Fp1–Fp2) and occipital (O1–O2) channel, see Fig. 2. Therefore, our results demonstrate that cross-channel FBA comparisons are valid and indeed capture true functional electrophysiological spatial maturation differences.

### 4.4 Clinical implications and future directions

Having established both the functional–structural divergence and the validity of our method, we now turn to clinical relevance. Quantifying spatial EEG maturation using machine learning may serve as a biomarker for preterm brain development and enable early identification of infants at risk for neurodevelopmental impairments, similar to FBA(Ansari et al., 2024; Mader et al., 2024; Pillay et al., 2020; Stevenson et al., 2020). Integrating automated sd_FBA estimation into routine NICU EEG monitoring could provide real-time insights into neurodevelopmental progression, guiding early interventions. The observed frontal lead and the subsequent central catchup emerged at the cohort level, underscoring the need for longitudinal follow-up studies to validate findings and explore individual variability.

### 4.5 Strengths and limitations

A major strength of this study is the use of a prospectively collected, longitudinal preterm EEG dataset with recordings starting before 26 weeks PMA combined with channel-specific deep learning models that achieved high accuracy and demonstrated strong cross-channel consistency. Nevertheless, several limitations should be acknowledged. Although we identified clear sd_FBA trajectories, the precise neurophysiological mechanisms underlying these differences remain incompletely understood. Reduced FBA prediction accuracy toward the boundaries of the training age range may have influenced the magnitude of age-dependent changes in spatial gradients. However, the consistent frontal–central–occipital ordering across PMA groups, including at the age boundaries, suggests that model performance alone is unlikely to explain the observed spatial organization (Fig. 2, Fig. 4). All EEG recordings were performed on preterm infants, this means that these results are only representative for ex-uterine- and not in-uterine development, which could be different. In addition, the sample size was insufficient to fully characterize individual longitudinal trajectories or to robustly link spatial maturation patterns to later cognitive outcomes. Addressing these questions will require larger cohorts with extended follow-up and multimodal integration of EEG with structural and functional imaging.

## 5 Conclusion

We introduced the sd_FBA metric as a novel approach to quantify regional functional development in preterm infants. Across datasets, sd_FBA revealed a reproducible frontal-occipital gradient, with frontal regions leading, central regions catching up toward term, and a delay in occipital regions. Additionally, there is also a temporal-central gradient with higher functional maturation in the central channel. These findings suggest that functional dynamics emerge earlier in the frontal cortex than structural imaging alone would predict. By providing a normalized and PMA-independent index, sd_FBA offers a new tool to study functional maturation and its spatial organization. Future work should assess whether deviations in sd_FBA trajectories are linked to neurodevelopmental outcomes, thereby establishing its translational value.

## CRediT authorship contribution statement

**Johannes Mader:** Writing – review & editing, Writing – original draft, Visualization, Validation, Project administration, Methodology, Formal analysis, Data curation, Conceptualization. **Manfred Hartmann:** Writing – review & editing, Supervision, Methodology, Formal analysis, Conceptualization. **Katrin Klebermass-Schrehof:** Writing – review & editing, Data curation. **Tobias Werther:** Writing – review & editing, Data curation. **Anastasia Dressler:** Writing – review & editing, Methodology, Data curation. **Johannes Koren:** Writing – review & editing, Data curation. **Gregor Kasprian:** Writing – review & editing, Conceptualization. **Victor Schmidbauer:** Writing – review & editing. **Renate Fuiko:** Writing – review & editing, Data curation. **Lisa Oberdorfer:** Writing – review & editing, Data curation. **Nadine Pointner:** Writing – review & editing, Methodology, Data curation. **Angelika Berger:** Writing – review & editing, Data curation. **Tilmann Kluge:** Writing – review & editing, Supervision. **Vito Giordano:** Writing – review & editing, Supervision, Methodology, Formal analysis, Data curation, Conceptualization.

## Contributors’ statement

V.G., M.H., and J.M. conceptualized and designed the study. J.M. performed the mode building, training, and evaluation. J.M. performed statistics, created all figures, and wrote the first draft of the manuscript.

V.G. coordinated and supervised EEG data collection. R.F. coordinated and supervised cognitive outcome data collection. V.G., A.B., K.K., L.O., A.D., N.P., J.K., and T.W. were involved in recording and analyzing the EEG data and contributed important suggestions regarding medical and scientific aspects and critical revision to the final version of the manuscript.

G.K. and V.S. contributed to situating the findings of this study within the context of structural brain maturation.

M.H. and T.K. contributed with important suggestions regarding technical aspects to the final version.

All authors reviewed the manuscript.

## Statement of ethics

This study was approved by the local ethics committee (EK Nr: 67/2008) of the Medical University of Vienna.

## Patient consent statement

Patient informed consent was obtained from all patients.

## Declaration of Generative AI and AI-assisted technologies in the writing process

AI-based tools were used to support language editing and improve clarity of the manuscript text. All scientific content, data analysis, interpretation of results, and final decisions regarding wording and conclusions were performed by the authors, who take full responsibility for the manuscript. The illustration of the infant’s head in the graphical abstract was created using a generative AI–assisted image tool and was subsequently reviewed and edited by the authors.

## Funding/support

This study was partially supported by the 10.13039/501100002428FWF (Dr Gottfried and Dr Vera Weiss Wissenschaftsstiftung), project number: KLI 1016-B. For open access purposes, the author has applied a CC BY public copyright license to any author accepted manuscript version arising from this submission.

## Declaration of Competing Interest

The authors declare that they have no known competing financial interests or personal relationships that could have appeared to influence the work reported in this paper.

## Data Availability

Data will be made available on request. All data generated or analyzed during this study are included in this published article (and its Supplementary Material files). The EEG datasets analyzed during the current study are not publicly available due to privacy policies at the medical university of Vienna but are available from the corresponding author on reasonable request.
