# Supplementary material for: Frontal lead in preterm functional brain maturation
Source: Dev Cogn Neurosci. 2026 Oct 2;82:101830. doi: 10.1016/j.dcn.2026.101830 (PMC13673055; doi:10.1016/j.dcn.2026.101830)
Supplement: Supplementary file 1 [file mmc1.docx]

Supplementary material

## A.1 Illustrative example for spatial EMA value

As an illustrative example for this method, consider a theoretically age-appropriate EEG at 30 weeks PMA, where each model predicts an EMA of 30 weeks for its corresponding channel. Now imagine the frontal model predicts the occipital channel to be 28 weeks EMA, while the occipital model predicts the frontal channel to be 32 weeks EMA. This implies that the occipital EEG at 30 weeks resembles the frontal EEG at 28 weeks, and the frontal EEG at 30 weeks resembles the occipital EEG at 32 weeks. In other words, the frontal EEG is estimated to be 2 weeks ahead of the occipital EEG, since the occipital pattern corresponds to what the frontal region displayed two weeks earlier.

## A.2: CNN models, training and evaluation

EEG maturational age was estimated using an end-to-end convolutional neural network (CNN) that processes raw EEG segments directly, without manual feature extraction. The architecture was closely based on the medium CNN model described in our previous work (Mader et al., 2026), with the key modification that only a single EEG channel was used as input instead of multiple channels. Apart from this adaptation, the architectural design principles and training strategy remained unchanged.

All models used a receptive field of 144 seconds. The architectures consisted of convolution–pooling (conv-pool) blocks that reduced the temporal resolution from 64 Hz to 1 Hz. Valid padding was used throughout to avoid edge artefacts. Max pooling was applied except in the final block, which used average pooling. Kernel and pooling sizes were chosen such that each output had a receptive field of exactly 144 seconds.

During training we used data augmentation, including Gaussian noise (SD 0.5), random scaling (0.8–1.2), horizontal flipping (50% probability), and random jitter (magnitude 0.4).

The network has two 1D-convolutional input conv-pool blocks with kernel-sizes of 65 and 9 followed by three 1D-conv-pool blocks and one final convolutional layer. It has in total 53 793 trainable parameters. The model comprises two parallel input branches: one employing a wide convolutional kernel to capture frequency-related features, and the other using a shorter kernel to extract local temporal features. The final convolutional-pooling layer applies average pooling to reduce the temporal resolution to 1 Hz. Subsequently, a fully connected layer integrates the extracted features to generate one prediction for each one-second interval. Convolutional kernels of different temporal extents allow the model to capture both short-term transient patterns (e.g. bursts, sharp amplitude changes) and longer-term rhythmic modulations associated with EEG maturation. Pooling layers reduce dimensionality and provide temporal invariance, improving robustness against local temporal variability.

Each model is trained to regress postmenstrual age (PMA), under the assumption that in infants without major neurological complications and with normal neurodevelopmental outcome, PMA provides a valid proxy for functional brain maturity.

Model training and evaluation were performed using 8-fold cross-validation, ensuring that recordings from the same infant were never split across training and validation folds. This procedure reduces overfitting and provides an unbiased estimate of generalization performance. Within each fold, the model was trained using the Adam optimizer with mean squared error as the loss function. EEG recordings of variable duration were segmented internally by the model, and per-second age estimates were aggregated by their median to obtain a single FBA value per recording.

The use of a single-channel CNN was essential for the spatial analyses performed in this study, as it allowed the training of channel-specific models with comparable architecture and performance. This ensured that spatial differences in predicted FBA reflected genuine differences in EEG maturation rather than architectural or input-dimensional biases. A detailed schematic of the original medium-sized CNN architecture, along with additional training parameters and evaluation procedures, is provided in Supplementary Material A1.

## A.3: Clustering of channels based on similarity of FBA estimations

To account for redundancy between channel-specific FBA estimators, EEG channels were grouped according to the similarity of their FBA predictions across all evaluated recordings. For each pair of channels, we computed the mean absolute difference between their estimated EEG maturational ages (EMA), averaged across all recordings for which predictions from both channel-trained models were available. This resulted in a symmetric channel-by-channel distance matrix, where smaller values indicate greater similarity in maturational behaviour between the corresponding channels.

Prior to clustering, the distance matrix was standardized using z-score normalization to ensure comparable scaling across channels. Channels were then grouped using the K-means clustering algorithm, applied to the normalized distance profiles. The optimal number of clusters was determined using the elbow method, based on the within-cluster sum of squared distances (inertia), which indicated a clear inflection point at three clusters. Accordingly, clustering was performed with K=3.

After clustering, cluster labels were reordered according to the median intra-cluster mean distance to impose a consistent ordering across analyses. The resulting clusters reflect groups of channels with similar FBA characteristics, rather than anatomical proximity. The clustered distance matrix is visualized as a heatmap in Figure 1b.

## A.4: Comparison between the sd_FBA values and the ΔFBA values from Stevenson et al. 2025.

Our sd_FBA values and the ΔFBA values calculated by Stevenson et al. (2025) are similar but have small differences. Let $\mathrm{FBA}_{chE,chT}$ be the FBA estimation of the channel chE from the model trained on channel chT. The metric ΔFBA is defined by

$$\Delta FBA_{ch}:=\frac{1}{n-1}\sum_{ch_{E}\neq ch} FBA_{ch_{E},\mathrm{ch}}-FBA_{ch,ch}$$

describes the average difference in FBA estimation based only on the model from channel ch.

Although both ΔFBA and sd_FBA aim to characterize spatial differences in functional brain maturation, they differ fundamentally in their interpretation and the question they address. ΔFBA is a model-centric metric, which quantifies how a model trained on a specific channel generalizes to other channels relative to its own training channel. It therefore reflects the directional transferability of maturational features encoded in that channel: a positive ΔFBA indicates that maturational patterns learned from this channel tend to predict higher FBA in other channels than in itself, suggesting that this channel exhibits an advanced or leading maturational signature.

In contrast, sd_FBA is a signal-centric metric, which quantifies how mature the EEG signal of a given channel appears relative to all other channels within the same recording, after accounting for model redundancy via clustering and normalization. sd_FBA therefore reflects the instantaneous relative maturity of a channel at a given developmental stage, independent of which channel provides the maturational template.

In summary, ΔFBA captures asymmetric maturational gradients across channels driven by model generalization, whereas sd_FBA captures within-recording spatial maturity differences of the EEG itself. Consequently, ΔFBA is best interpreted as a population-level indicator of spatial maturation hierarchy, while sd_FBA provides a recording-level, age-independent measure of regional maturational advancement or delay.

F-O and T-C trends for ΔFBA were similar to those with sd_FBA but weaker.

### A.4.1Frontal–occipital gradient in ΔFBA

Across the full PMA range, ΔFBA showed a clear anterior–posterior organization along the frontal–occipital (F–O) axis (r = −0.49, p < 0.001), with frontal channels exhibiting the highest ΔFBA values and occipital channels the lowest (Supplementary Fig. A1, first row).

#### Figure A1: Temporal evolution of the frontal-occipital and temporal-central gradient in ΔFBA for four different PMA groups

*First row: Fronto–occipital gradient of difference in functional brain age (*Δ*FBA) across postmenstrual age (PMA) groups. Boxplots show* Δ*FBA distributions for frontal (F), frontal-central (F–C), central (C), central-occipital (C–O), and occipital (O) channel within four PMA ranges. Second row: Temporal-central gradient of ΔFBA in the four age groups. Boxplots show ΔFBA distributions for the T3-C3, C3-C4, and C4-T4 channel. Orange lines indicate linear trends across the frontal-occipital axis or the lateral. Pearson correlation coefficients (r) and corresponding p-values are reported in each panel.*

When examining individual recordings, 91.9% of all F–O gradients were negative, with a median gradient of −0.42 (IQR [−0.65, −0.22]; Supplementary Fig. A2). Furthermore, individual F–O gradients became more negative with increasing PMA (r = −0.23, p = 0.001; Supplementary Fig. A2), indicating that the directional maturational hierarchy captured by ΔFBA becomes increasingly pronounced with advancing postmenstrual age.

### A.4.2Temporal–central gradient in ΔFBA

Along the lateral axis, ΔFBA exhibited a weaker and less consistent spatial organization. Across the full PMA range, a modest positive correlation was observed (r = 0.35, p < 0.001), reflecting higher ΔFBA values for central (C3–C4) compared with lateral (T3–C3 and C4–T4) channels (Supplementary Fig. X, second row).

#### Figure A2: Temporal evolution of the frontal-occipital and temporal-central gradient in ΔFBA for four different PMA groups

*First row: Frontal–occipital gradient of spatial difference in functional brain age (*Δ*FBA). Left) Boxplots show* Δ*FBA distributions for frontal (F), frontal-central (F–C), central (C), central-occipital (C–O), and occipital (O) channel for all recordings with a negative correlation of r=-0.64. Right) Evolution of individual F-O-gradients per PMA in weeks. Second row: Temporal-central gradient of* Δ*FBA. Left) Boxplots show ΔFBA distributions for the T3-C3, C3-C4, and C4-T4 channel. Right) Evolution of individual T-C-gradients per PMA in weeks. Orange lines indicate linear trends across the frontal-occipital axis or the lateral. Pearson correlation coefficients (r) and corresponding p-values (p) are reported in each panel.*

At the level of individual recordings, temporal–central gradients were predominantly positive, with a median gradient of 0.60 (IQR [0.01, 1.31]; Supplementary Fig. A2). However, the association between individual T–C gradients and PMA was weak and did not reach statistical significance (r = 0.19, p = 0.005; Supplementary Fig. A2). This suggests that, unlike the robust frontal–occipital pattern, lateral maturation gradients captured by ΔFBA are less systematically modulated by developmental stage.

Overall, while ΔFBA consistently reveals a strong and PMA-dependent frontal–occipital maturation hierarchy, lateral gradients appear secondary and more variable, underscoring the axis-specific sensitivity of model-based maturation gradients.

## A.5: Complementary replication on an external dataset

We calculated the spatial EEG-maturation on the EEG seizure dataset from Helsinki (Stevenson et al., 2019).

The Helsinki dataset consisted of multichannel EEGs from 79 infants with a focus on seizure events. In the EEG of 22 infants, there was no seizure annotated, and in the EEG from 10 infants only one out of 3 reviewers annotated a seizure. We used the data from these 32 infants but excluded the annotated seizure events.

The remaining 32 EEGs were from 5 preterm infants, 26 term infants, and one infant without information on GA. EEG recordings were performed between 36 and 44 weeks of PMA.

This dataset consisted of multichannel EEG recorded on 19 electrodes, as shown in Fig. A1.

*Figure A3: Electrode position of Helsinki dataset*

*Electrodes of the Helsinki dataset were positioned according to the international 10-20 system, positions coloured in red were also present in the original analysis, all other were coloured in violet.*

sd_FBA was calculated similar to the PREMOS dataset. Since this dataset contained more channels, we first calculated the channel-wise EMA for each of the 19 channels as the weighted mean of the 13 model predictions, using the clustering derived from the training dataset. Next, we calculated a new clustering of the 19 channels based on the channel-wise EMA values. Then the sd_FBA was calculated as the EMA minus the weighted average of the EMA values of the same recording.

The new clustering led again to 3 clusters, a frontal, central, and occipital one. These can be seen in the Fig. below. The temporal and spatial evolution of the sd_FBA is comparable to the PREMOS dataset.

*Figure A4: Visualisation of the sd_FBA values calculated from the Helsinki dataset.*

*Visualization of the temporal evolution of the spatial EEG age difference (sd_FBA) in two groups. The first row shows Topo-plots of the spatial maturation values in two PMA groups. The second row shows the corresponding boxplots of the three clusters.*

## A.6: Complementary replication with an externally trained model

To further enforce our results, we tried to replicate the same results with a single channel EMA-estimation algorithm trained on a completely different dataset. This dataset consisted of EEG recorded on aEEG monitors at the Comprehensive Center of Pediatrics, Medical University of Vienna, between 2014 and 2022, with approval from the local ethics committee (EK Nr: 1814/2018). We refer to this dataset as D2, and to the EEG dataset used in the main manuscript as D1.

This EEG dataset was acquired using Olympic Brainz Monitors (Natus®, San Carlos, USA) with four active electrodes positioned at C3, C4, P3, and P4. EEG was collected at a sampling frequency of 200 Hz.

sd_FBA was calculated similar to the original analysis. We used the three single-channel models trained on the channels C3-C4, C3-P4, P3-P4 as an ensemble model and calculated the EMA of each channel as the mean of all tree channels. In this case the EMA is equal to the SFBA, since there is only one estimation model. Next, we calculated a new clustering of the 13 channels based on the similarity in SFBA values. The new clustering led again to 3 clusters, a frontal, central, and occipital one, which was slightly different to the clustering in the main manuscript. In this new clustering, the channels Fp1-T3 and Fp2-T4 both were in the central cluster instead of the temporal-occipital cluster, as shown in Fig. A3. Then the sd_FBA was then calculated as before as the SFBA minus the weighted average of the SFBA values of the same recording. The temporal and spatial evolution of the sd_FBA values in this case is comparable to results in our main study.

#### Figure A5: sd_FBA values visualised at different PMA ranges.

*Visualization of the temporal evolution of the relative spatial EEG maturation(sd_FBA) in four groups. The first row shows Topo-plots of the sd_FBA values in four PMA groups. The second row shows the corresponding sd_FBA trend of the three clusters.*

## A.7 sd_FBA based on monopolar montage

In addition to the bipolar montage used for the main analysis, we repeated the complete analysis using a monopolar EEG montage to assess whether the observed spatial maturation patterns depended on the choice of EEG derivation. The monopolar montage comprised the eight channels Fp1, Fp2, C3, C4, T3, T4, O1, and O2. Importantly, this analysis was not based on a re-referencing or transformation of the FBA estimates obtained from the bipolar analysis. Instead, the entire analytical pipeline was repeated independently for the monopolar montage, starting from the pre-processed monopolar EEG signals. Separate single-channel CNN models were trained for each of the eight monopolar channels to estimate FBA, following the same model architecture, training procedure, and cross-validation strategy as used for the bipolar analysis. Each independently trained monopolar model was subsequently evaluated on all monopolar channels, and channel similarities, clustering, sFBA, and sd_FBA were recalculated based exclusively on these monopolar FBA estimates. The subsequent analyses of spatial maturation, including cluster-wise developmental trajectories and the frontal–occipital and temporal–central gradients, were likewise repeated using the monopolar sd_FBA values. Thus, the monopolar analysis represents an independent analysis of the methodological pipeline rather than a simple alternative representation of the bipolar results.

### A.7.1 Clustering of similarly evolving channels

The elbow-method suggested again three clusters. The first cluster consisted of only the frontal channel *Fp1 and Fp2*. The second cluster included the central channels *C3 and C4.* The third cluster included all other channels. Similarly to the bipolar case, we refer to them as the frontal cluster (F), central cluster (C), and temporal-occipital cluster (TO). The positions of these clusters are visualised in Fig. 3 and similarities of all channel pairs are visualized in Fig. B.2.

#### Figure A6: The process of calculating the sd_FBA value.

*a) All single-channel models are evaluated on all channels. b) Heatmap visualizing channel-similarity in FBA estimation and clustering. c) Spatial location of the three clusters. d) An example of FBA for the channel Fp1-Fp2 from all single-channel models and the sFBA values as the weighted average. PMA at time of recording was 31+0 weeks. e) sFBA values for all channels and the sd_FBA values as the differences between the sFBA and the weighted average for the same recording as d). f) Topoplot visualizing the sd_FBA values from e) spatially.*

### A.7.2. Spatial differences in FBA

sd_FBA values, measuring the relative FBA of one channel compared to all others, were calculated from 93 recordings. 99% of sd_FBA values ranged between -1.85 and 1.72. The frontal channels Fp1 and Fp2 had the highest median value at 0.22 and 0.24. The temporal channels T3 and T4 had the lowest median sd_FBA values of -0.45 and -0.38. This distribution of sd_FBA values is in accordance with the channel wise comparison in Fig. A7.

#### Figure A7: Monopolar pair wise PMA vs FBA

*Visualization of PMA values vs. FBA values for all channel pairs of the channels Fp2, C4, and O2. The labelling “Ev. F, Tr. C”, for example, indicates FBA values for the frontal evaluation channel Fp2 by the model trained on the central channel C4.*

#### Table A1: Distribution of monopolar sd_FBA values across all channels

|  | Median | Min | Q1 | Q3 | Max | p0.5 | p99.5 | IQR |
| --- | --- | --- | --- | --- | --- | --- | --- | --- |
| all | -0.08 | -3.17 | -0.47 | 0.30 | 2.73 | -1.85 | 1.72 | 0.77 |
| Fp1 | 0.22 | -0.96 | -0.05 | 0.54 | 2.07 | -0.71 | 1.56 | 0.59 |
| Fp2 | 0.24 | -1.17 | -0.04 | 0.52 | 2.01 | -0.85 | 1.98 | 0.56 |
| C3 | 0.04 | -3.17 | -0.30 | 0.43 | 2.16 | -2.03 | 1.68 | 0.73 |
| C4 | 0.05 | -2.62 | -0.27 | 0.49 | 1.86 | -1.84 | 1.45 | 0.77 |
| T3 | -0.45 | -2.61 | -0.75 | -0.11 | 0.92 | -2.20 | 0.73 | 0.65 |
| T4 | -0.38 | -2.01 | -0.70 | -0.06 | 1.56 | -1.80 | 1.36 | 0.64 |
| O1 | -0.20 | -1.85 | -0.56 | 0.12 | 2.73 | -1.77 | 1.96 | 0.68 |
| O2 | -0.32 | -2.06 | -0.62 | 0.01 | 1.41 | -1.94 | 1.02 | 0.63 |

Median, minimum, first quartile, third quartile, maximum, and interquartile range for all channels.

### A.7.3. Evolution of spatial maturation with PMA

The median sd_FBA values within each PMA group were visualised in Topoplots which capture the spatial component of these values, see Fig. A8. The grouped monopolar sd_FBA values were symmetric from left to right across all age groups but showed a shift from frontal to temporal-occipital regions.

In the earliest age group (25–27+6 weeks PMA), monopolar sd_FBA values were higher in the frontal cluster compared to the other clusters (p < 0.003 for both). As PMA increased, sd_FBA values in the central channels increased. Values in both frontal and central clusters remained significantly higher than those in the occipital cluster (p < 0.001) between 28 weeks PMA and 35+6 weeks PMA.

#### Figure A8: Monopolar sd_FBA values spatially visualised at different PMA ranges.

*Visualization of the temporal evolution of the spatial difference in FBA (sd_FBA) in four groups. The first row shows Topoplots of the spatial maturation values in four PMA groups. The second row shows the corresponding spatial maturation trend of the three clusters.*

### A.7.4. Gradients in monopolar sd_FBA

Across the full PMA range, sd_FBA exhibited a pronounced frontal-occipital gradient along the frontal–occipital (F–O) axis (r = −0.36, p < 0.001), with frontal channels showing the highest relative maturity and occipital channels the lowest, see Fig. A9 (first row). When stratified by PMA, this gradient was lower at early ages (25–28 weeks PMA; r = −0.30, p < 0.001) and the largest with 28–32 weeks (r = −0.47, p < 0.001).

79.2% of all individual F-O-gradients are negative with a median gradient of -0.23 (IQR [-0.47, -0.04]). The consistent increase in correlation indicates an increasingly well-defined spatial ordering of FBA along the frontal-occipital axis with advancing PMA.

Along the lateral axis, monopolar sd_FBA showed a stronger spatial organization. Across the full PMA range, a modest positive correlation was observed (r = 0.39, p < 0.001), reflecting higher sd_FBA in central compared with temporal channels, see Fig. A9 (second row). Age-stratified analyses showed a significant lateral gradient already at the youngest ages (25–28 weeks PMA; r = 0.31, p < 0.001), which was strongest between 28- and 32-weeks PMA (r = 0.58, p < 0.001). The gradient subsequently weakened at 32–36 weeks PMA (r = 0.34, p < 0.001) and was no longer evident at 36–41 weeks PMA (r = 0.09, p = 0.332). Thus, the temporal–central organization was most pronounced during early-to-intermediate preterm development and progressively diminished toward term-equivalent age.

Individual T-C-gradients are mostly positive (76%) with a median gradient of 0.42 (IQR [0.01, 0.91]).

#### Figure A9: Temporal evolution of the frontal-occipital and temporal-central gradient in sd_FBA for four different PMA groups

*First row: Fronto–occipital gradient of spatial difference in functional brain age (sd_FBA) across postmenstrual age (PMA) groups. Boxplots show sd_FBA distributions for frontal (F), frontal-central (F–C), central (C), central-occipital (C–O), and occipital (O) channel within four PMA ranges. Second row: Temporal-central gradient of sd_FBA in the four age groups. Boxplots show sd_FBA distributions for the T3, C3, C4, and T4 channel. Orange lines indicate linear trends across the frontal-occipital axis or the lateral. Pearson correlation coefficients (r) and corresponding p-values are reported in each panel.*

### A.7.5 Comparison with the bipolar montage

Overall, the monopolar analysis closely reproduced the principal findings obtained with the bipolar montage. Both approaches identified a comparable spatial organization of functional brain maturation, with relatively advanced frontal maturation, followed by central regions, and lower sd_FBA values in temporal and occipital regions. In both montages, the clustering analysis yielded three spatially comparable groups, and the developmental analyses demonstrated systematic frontal–occipital as well as temporal–central gradients. Although the magnitude and age-specific strength of these gradients differed between the two montages, their overall direction and developmental pattern were consistent. In particular, both analyses supported a frontal lead in functional maturation and a relative delay of posterior regions, while the lateral temporal–central organization was less pronounced and more age-dependent. Thus, the main findings of this study were robust to the choice of EEG derivation and cannot be explained by the use of a bipolar montage alone. The monopolar analysis therefore provides a complementary sensitivity analysis supporting that sd_FBA captures spatial differences in functional brain maturation that are preserved across different EEG montage configurations.

## B. Additional tables and figures

#### Table B.1: Channel pairwise FBA estimation

|  | **Fp1-Fp2** | **C3-C4** | **O1-O2** | **Fp1-C3** | **Fp2-C4** | **Fp1-T3** | **Fp2-T4** | **T4-C4** | **T3-C3** | **C3-O1** | **C4-O2** | **T3-O1** | **T4-O2** | **av** |
| --- | --- | --- | --- | --- | --- | --- | --- | --- | --- | --- | --- | --- | --- | --- |
| **Fp1-Fp2** | 100% | 87% | 100% | 93% | 89% | 97% | 95% | 97% | 99% | 97% | 98% | 98% | 99% | 96% |
| **C3-C4** | 13% | 100% | 93% | 80% | 64% | 93% | 87% | 83% | 89% | 82% | 87% | 85% | 83% | 78% |
| **O1-O2** | 0% | 7% | 100% | 5% | 5% | 29% | 31% | 26% | 34% | 9% | 14% | 45% | 33% | 20% |
| **Fp1-C3** | 7% | 20% | 95% | 100% | 30% | 89% | 74% | 61% | 76% | 76% | 86% | 77% | 76% | 64% |
| **Fp2-C4** | 11% | 36% | 95% | 70% | 100% | 87% | 86% | 78% | 81% | 82% | 89% | 81% | 85% | 74% |
| **Fp1-T3** | 3% | 7% | 71% | 11% | 13% | 100% | 33% | 16% | 19% | 42% | 61% | 50% | 49% | 31% |
| **Fp2-T4** | 5% | 13% | 69% | 26% | 14% | 67% | 100% | 34% | 40% | 52% | 70% | 52% | 63% | 42% |
| **T4-C4** | 3% | 17% | 74% | 39% | 22% | 84% | 66% | 100% | 49% | 58% | 65% | 72% | 70% | 52% |
| **T3-C3** | 1% | 11% | 66% | 24% | 19% | 81% | 60% | 51% | 100% | 47% | 57% | 75% | 66% | 47% |
| **C3-O1** | 3% | 18% | 91% | 24% | 18% | 58% | 48% | 42% | 53% | 100% | 59% | 72% | 67% | 46% |
| **C4-O2** | 2% | 13% | 86% | 14% | 11% | 39% | 30% | 35% | 43% | 41% | 100% | 58% | 60% | 36% |
| **T3-O1** | 2% | 15% | 55% | 23% | 19% | 50% | 48% | 28% | 25% | 28% | 42% | 100% | 42% | 31% |
| **T4-O2** | 1% | 17% | 67% | 24% | 15% | 51% | 37% | 30% | 34% | 33% | 40% | 58% | 100% | 34% |

Percentage of how often the column channel model estimated the row channel higher than vice versa.

#### Table B.2: Distribution of sd_FBA values across all channels

|  | **Median** | **Min** | **Q1** | **Q3** | **Max** | **IQR** |
| --- | --- | --- | --- | --- | --- | --- |
| **Fp1-Fp2** | **0.64** | -1.23 | **0.27** | **1.04** | 3.09 | 0.77 |
| **Fp1-C3** | 0.02 | -2.74 | -0.32 | 0.40 | 1.63 | 0.72 |
| **Fp2-C4** | 0.17 | -1.25 | -0.28 | 0.49 | 2.23 | 0.77 |
| **C3-C4** | **0.05** | -2.24 | **-0.25** | **0.52** | 2.69 | 0.78 |
| **Fp1-T3** | -0.60 | -3.04 | -1.05 | -0.22 | 2.14 | 0.83 |
| **Fp2-T4** | -0.50 | -2.22 | -0.95 | -0.05 | 1.66 | 0.90 |
| **T4-C4** | -0.46 | -4.43 | -0.99 | -0.07 | 1.52 | 0.93 |
| **T3-C3** | -0.54 | -4.76 | -0.99 | -0.17 | 1.21 | 0.81 |
| **C3-O1** | -0.72 | -2.62 | -1.15 | -0.28 | 1.25 | 0.87 |
| **C4-O2** | -0.69 | -3.11 | -1.17 | -0.33 | 1.95 | 0.84 |
| **T3-O1** | -0.94 | -2.74 | -1.51 | -0.54 | 1.08 | 0.97 |
| **T4-O2** | -0.89 | -3.10 | -1.49 | -0.43 | 0.59 | 1.06 |
| **O1-O2** | **-0.97** | -5.18 | **-1.54** | **-0.55** | 1.11 | 0.99 |
| **temporal** | **-0.49** | -4.76 | **-0.99** | **-0.11** | 1.52 | 0.88 |

Median, minimum, first quartile, third quartile, maximum, and interquartile range for all channels. Temporal is the average between the channels T3-C3 and T4-C4.

#### Figure B.1: Evaluation of each single-channel model

*Boxplots of PAD for each of the 13 bipolar channel models evaluated on their testing data from the 8-fold cross validation.*

#### Figure B.2: Boxplots of sd_FBA values separated by cluster for four different PMA group

*Visualization of the temporal evolution across four age groups of the spatial difference in FBA (sd_FBA) of the three clusters in Boxplots. Bars above the boxplots indicate significant differences in mean (p<0.001).*

#### Figure B.3: Temporal evolution of the frontal-occipital and temporal-central gradient in sd_FBA for four different PMA groups

*First row: Frontal–occipital gradient of spatial difference in functional brain age (sd_FBA). Left) Boxplots show sd_FBA distributions for frontal (F), frontal-central (F–C), central (C), central-occipital (C–O), and occipital (O) channel for all recordings with a negative correlation of r=-0.64. Right) Evolution of individual F-O-gradients per PMA in weeks. Second row: Temporal-central gradient of sd_FBA. Left) Boxplots show sd_FBA distributions for the T3-C3, C3-C4, and C4-T4 channel. Right) Evolution of individual T-C-gradients per PMA in weeks. Orange lines indicate linear trends across the frontal-occipital axis or the lateral. Pearson correlation coefficients (r) and corresponding p-values (p) are reported in each panel.*
